# Whole-Genome Sequence of the Mycoplasma mucosicanis Type Strain

**DOI:** 10.1128/MRA.00799-19

**Published:** 2019-10-10

**Authors:** Rebecca L. Tallmadge, Patrick K. Mitchell, Renee Anderson, Rebecca Franklin-Guild, Laura B. Goodman

**Affiliations:** a Animal Health Diagnostic Center, Cornell University, Ithaca, New York, USA; Indiana University, Bloomington

## Abstract

Whole-genome sequencing of Mycoplasma mucosicanis type strain 1642 was performed to support efforts to better understand the clinical significance of Mycoplasma infection in canine health. The availability of this sequence will also further the development of highly specific diagnostic tests.

## ANNOUNCEMENT

Dogs are susceptible to multiple Mycoplasma species, although not all *Mycoplasma* infections cause clinical disease (1, 2). Mycoplasma mucosicanis strain 1642^T^ was isolated from the genital mucosa of a healthy male dog, and additional isolates were recovered from the oral cavity or genital tract of other healthy dogs (3). Given that *M. mucosicanis* was initially described in 2011 and to date has been characterized only in healthy dogs, its impact on canine health is not known (3). Prior to this report, publicly available sequence information for *M. mucosicanis* was limited to *rpoB* and 16S, 23S, and the 16S-23S intergenic spacer region sequences.

The *M. mucosicanis* 1642^T^ strain (ATCC BAA-1895) was purchased (American Type Culture Collection, Manassas, VA), subcultured onto modified 9CFR *Mycoplasma* agar, and grown in Hardy Diagnostics *Mycoplasma* broth medium (Santa Maria, CA) for 2 days at 33 to 37°C with 6% CO_2_. DNA was extracted from culture broth with the MagMAX core nucleic acid purification kit (Applied Biosystems, Foster City, CA). DNA concentration was quantified with a Qubit 3.0 fluorometer (Thermo Fisher Scientific, Waltham, MA). The Nextera XT DNA library preparation kit (Illumina, San Diego, CA) was used to generate a tagmented library, which was purified using AMPure XP beads (0.5× concentration; Beckman Coulter Life Sciences, Indianapolis, IN). This library was pooled with others and run on an Illumina MiSeq reagent kit (V3) cartridge at 2 × 250 cycles using a MiSeq sequencer. Sequence read quality metrics were calculated using FastQC 0.11.8 (https://www.bioinformatics.babraham.ac.uk/projects/fastqc/). We produced 150,886 reads with average lengths of 224.25 bp and 224.24 bp and average Q scores of 36.48 and 36.20 for the forward and reverse reads, respectively. Reads were trimmed using the sliding window program in Trimmomatic 0.36, with a window size of 4 and quality threshold of 20 (4). Trimmed reads were assembled using SPAdes 3.10.1, with automatic k-mer selection and the careful option enabled (5). Contigs shorter than 200 bp were excluded. The initial draft genomes were submitted to the NCBI, and contigs flagged for possible contamination were removed. Read depth and assembly statistics were calculated using BBMap 38.26 (https://jgi.doe.gov/data-and-tools/bbtools/) and QUAST 4.0 (6). The *M. mucosicanis* type strain 1642 (ATCC BAA-1895) assembly consisted of 869,512 bp, with 57 contigs, an *N*_50_ value of 64,609 bp, and a GC content of 29.54%. The average read depth was 77.4×.

This whole-genome sequence is intended to further the understanding of the role of *Mycoplasma* infection in canine disease, as an individual pathogen and as a contributor to coinfections with other bacteria and viruses (7).

### Data availability.

The *M. mucosicanis* 1642^T^ whole-genome sequence described here is associated with NCBI BioProject number PRJNA525414. The raw reads are available in the Sequence Read Archive (accession number SRR8689604). This project has been deposited at DDBJ/ENA/GenBank under the accession number SMDN00000000; this paper describes version SMDN01000000. The GenBank assembly accession number for *M. mucosicanis* 1642^T^ is GCA_006546935.
